# Diagnosis and Management of Takotsubo Syndrome in Acute Aneurysmal Subarachnoid Hemorrhage: A Comprehensive Review

**DOI:** 10.31083/j.rcm2406177

**Published:** 2023-06-19

**Authors:** Dorottya Szántó, Péter Luterán, Judit Gál, Endre V. Nagy, Béla Fülesdi, Csilla Molnár

**Affiliations:** ^1^Department of Anesthesiology and Intensive Care, Faculty of Medicine, University of Debrecen, 4032 Debrecen, Hungary; ^2^ELKH-DE Cerebrovascular Research Group, 4032 Debrecen, Hungary; ^3^Department of Internal Medicine, Faculty of Medicine, University of Debrecen, 4032 Debrecen, Hungary

**Keywords:** Takotsubo syndrome, subarachnoid hemorrhage, neurogenic stunned myocardium, delayed cerebral ischemia, intracranial aneurysm treatment

## Abstract

Takotsubo syndrome (TS) is a frequent complication of subarachnoid hemorrhage (SAH), especially in massive SAH with severe neurological damage. The initial presentation of TS is similar to acute coronary syndrome, causing differential diagnostic issues. Unnecessary diagnostic steps and uncertainty in therapy may delay the definitive treatment of the aneurysm, therefore increasing the risk of rebleeding. The purpose of this review is to summarize the latest knowledge on the diagnosis and therapy of TS in SAH and to provide a diagnostic and therapeutic algorithm for the acute phase, promoting the early definitive treatment of the aneurysm. Rapid hemodynamic stabilization and early aneurysm securing are key points in reducing the risk of delayed cerebral ischemia and improving outcomes. In acute SAH noninvasive bedside diagnostic methods are preferred and securing the aneurysm is the priority. The combination of electrocardiography, cardiac biomarkers, and echocardiography is of great importance in differentiating TS from acute myocardial infarction. The risk-benefit ratio of coronary angiography should be carefully and individually considered and its use should be limited to patients with strong evidence of myocardial ischemia, after the successful endovascular treatment of the aneurysm. Invasive hemodynamic monitoring may be beneficial in cases of cardiogenic shock or pulmonary edema. In patients with hemodynamical instability secondary to TS, the use of non-catecholamine inotropes, especially levosimendan is recommended. In refractory hypotension, mechanical support should be considered. The left ventricular function improves within days to months after the acute event, low initial ejection fraction may predispose to delayed recovery.

## 1. Introduction 

Takotsubo syndrome (TS) is an acute, reversible cardiomyopathy, precipitated by intensive emotional or physical stressors. TS is also known as ‘stress cardiomyopathy’, ‘apical ballooning syndrome’, ‘Takotsubo cardiomyopathy’, ‘broken heart syndrome’, and ‘acute reversible myocardial injury’ [1]. The initial presentation of TS has similar features to acute coronary syndrome (ACS), however, coronary angiography usually shows an absence of significant coronary artery disease. It accounts for around 4% of patients presenting with ACS symptoms [2]. The pathomechanism of TS is not completely understood, but it seems to be related to catecholamine-induced myocardium stunning [3, 4]. Studies show a high incidence of TS in subarachnoid hemorrhage, up to 28% of patients have evidence of regional wall motion abnormalities (WMAs) on echocardiography, and up to 15% have global left ventricular dysfunction with depressed ejection fraction (EF) [2]. Complications of TS, such as cardiac arrhythmias and congestive heart failure, make the management of blood pressure and volume status challenging, especially in the setting of increased intracranial pressure or cerebral vasospasm [5]. Hemodynamic instability and differential diagnostic issues may delay surgical or endovascular treatment of the aneurysm, therefore rapid stabilization and avoidance of unnecessary diagnostic steps are key points in acute care.

The main purpose of this review is to summarize the latest knowledge on the diagnosis and treatment of TS in subarachnoid hemorrhage (SAH) and to provide a diagnostic and therapeutic algorithm for the acute phase, promoting the early definitive treatment of the aneurysm.

## 2. Search Strategy 

We reviewed the relevant literature, focusing mainly on research from the last 10 years, using PubMed. Our search terms were: “takotsubo cardiomyopathy”; “neurogenic stunned myocardium”; “takotsubo syndrome”; “stress-induced cardiomyopathy”; “takotsubo cardiomyopathy” and “subarachnoid hemorrhage”, “apical ballooning” and “subarachnoid hemorrhage”, “takotsubo syndrome” and “subarachnoid hemorrhage”, “stress-induced cardiomyopathy” and “subarachnoid hemorrhage”; “brain-heart crosstalk”.

## 3. Pathophysiology

The pathophysiology of TS is still incompletely understood. Recent research has drawn attention to the possible role of several molecular pathways, most of which involve catecholamine receptor signalling. The majority of the current knowledge is rooted in animal models, however, elevated circulating catecholamine level in TS patients has been confirmed by several human studies, as well [6, 7, 8].

The most common neurological disease associated with TS is SAH [2]. In SAH, rapidly progressing and severe intracranial hypertension may lead to overactivation of the sympathetic nervous system and high-dose catecholamine release (a maladaptive form of the Cushing reflex) [9]. The direct damage of cardiovascular centers (e.g., insula, hypothalamus, periaqueductal gray, brainstem) by the hemorrhage, delayed cerebral ischemia, epileptic seizures, hydrocephalus, and exogenous catecholamines may also be triggers of TS after SAH [7, 9, 10, 11]. Several studies have confirmed that plasma catecholamine level is elevated in SAH [5, 7, 12], however, an even higher serum level was demonstrated when SAH and TS were present together [13, 14]. In addition, our previous prospective study on SAH patients found higher normetanephrine concentration in the urine of patients with TS compared to control SAH patients, confirming the causative role of greater catecholamine release [15].

Elevated catecholamine levels can lead to myocardial stunning through several possible pathways. Three main hypotheses are often mentioned in the literature: epicardial coronary vasospasm, acute coronary microcirculatory dysfunction, and catecholamine-induced myocardial injury [1]. There is growing evidence of the presence of myocardial inflammation in the acute phase. Altered metabolism, certain genetic polymorphisms, and epigenetic changes have also been linked to TS [13, 16, 17]. Another important aspect is the higher frequency in postmenopausal women, which suggests the protective role of estrogen [16, 17, 18]. Excess catecholamine release and different pathophysiological pathways likely contribute to the development of TS to a varying degree in individual patients. Probably, the combination of an exaggerated sympathetic response along with an increase in myocardial sensitivity to catecholamines puts some patients at increased risk of developing TS [6, 19].

Catecholamine-induced myocardial injury mediated by β-adrenoreceptors (βAR) is the most established pathophysiological pathway. Epinephrine is a positive inotrope at low and modest levels via the β1AR-Gs signalling, however, at high levels, a switch of β2AR coupling from Gs to Gi causes a negative inotropic effect, a process called stimulus trafficking [20]. Excess activation of the β1AR-Gs pathway by the supraphysiological level of catecholamines, through activation of adenyl cyclase and cyclic adenosine monophosphate production, leads to activation of protein kinase A (PKA). PKA phosphorylates several substrates (e.g., L-type calcium channels, Ryanodine receptor 2, phospholamban, protein C) that play an important role in myocardial calcium homeostasis and contraction. This process leads to myocardial calcium overload, reduced calcium affinity of the myofilaments, mitochondrial dysfunction, oxidative stress, inflammation, apoptosis, and necrosis [3, 21]. Stimulus trafficking reduces this harmful effect of the β1AR-Gs pathway, switching to the antiapoptotic but at the same time negative inotropic β2AR-Gi pathway and leading to myocardial stunning and WMAs [3, 20].

Mammalian hearts demonstrate the highest density of βARs in the apex, reflecting the characteristic apical ballooning appearance of TS [20, 22, 23]. Atypical TS types include the midventricular, basal, and focal forms are generally less common, however, in acute neurological conditions appear to be more frequent [24]. The pathophysiological background of the atypical localization of wall motion abnormalities is not clearly understood. The altered distribution of the β2AR and higher local density of sympathetic nerve endings in the basal segments may provide a partial explanation [13, 25].

In patients with TS, WMA is often extensive enough to reduce left ventricular EF. According to previous studies, 13–17% of SAH patients have WMA in echocardiography with preserved EF, while 8–15% have a decreased EF even with symptoms of heart failure (e.g., low mean arterial pressure—MAP, cardiogenic shock, pulmonary edema) [13]. Patients with low EF have a higher risk for intraventricular thrombus formation and thromboembolism. Low MAP, especially when intracranial pressure is elevated or in the presence of cerebral vasospasm, can lead to a decrease in cerebral perfusion and consequent secondary damage to the brain. Arrhythmias and sudden cardiac death are also major complications of TS [22]. Neurogenic pulmonary edema (NPE) is known to be another consequence of direct catecholamine toxicity after cerebral insults. Catecholamine rush induces severe pulmonary vasoconstriction, resulting in raised hydrostatic pressure and increased permeability of the pulmonary capillaries. In these cases, cardiac function is almost always impaired, so it is difficult to distinguish between NPE and pulmonary edema secondary to acute heart failure [9, 26, 27].

## 4. Risk Factors

SAH is the most common neurological disease associated with TS [2]. It is not clearly understood why one patient develops TS and another with similar parameters does not. Most probably, a combination of individual predisposing factors and the degree of sympathetic stimulation is responsible for the appearance and severity of TS [28]. Some risk factors appear to influence the occurrence of TS in SAH, such as female gender, the extent of the hemorrhage, and the degree of neurological damage.

Most clinical trials have identified female gender as a risk factor for TS [11, 28, 29, 30, 31, 32]. Estrogen via receptor crosstalk inhibits signal transduction through βARs. Reduced estrogen level during menopause increases sympathetic drive and endothelial dysfunction, which may explain the higher incidence of TS in postmenopausal women. The difference in stress coping strategies in men and women may also contribute to sex disparity [16]. Several studies have found that patients with a higher Hunt-Hess or World Federation of Neurological Surgeons (WFNS) score, reflecting more severe neurological damage, have a higher risk of TS [28, 29, 30, 31, 33, 34, 35, 36]. Modified Fisher grade 3–4 (massive SAH with or without intraventricular bleeding) was also associated with TS [5, 15, 33]. A recent prospective study found that not only the occurrence but also the severity of TS was related to the modified Fisher score, the Hunt-Hess score, and the WFNS score [15]. Probably, an extensive hemorrhage with severe neurological damage triggers greater catecholamine release, leading to a higher risk of severe left ventricular dysfunction.

Regarding other risk factors such as posterior localization of the aneurysm and cerebral vasospasm, the available data are more contradictory [15, 26, 29, 30, 33, 37]. In contrast to acute myocardial infarction (AMI), age, and cardiovascular risk factors (hypertension, diabetes mellitus, obesity, hyperlipidemia, tobacco use) do not predispose to the development of TS [15, 33, 34, 35, 38]. Furthermore, advanced diabetes mellitus with autonomic neuropathy may have a protective effect [39].

## 5. Diagnosis 

The diagnosis of TS is often challenging because its clinical presentation resembles that of AMI. The first diagnostic criteria were introduced in 2003, which was followed by several others, the best known of which is the Mayo Clinic Diagnostic Criteria. In 2018, the InterTAK Diagnostic Criteria were created to improve diagnostic accuracy. These new criteria incorporate the most recent and updated evidence available for TS. Based on data from the International Takotsubo Registry, the InterTAK Diagnostic Score was also developed to help differentiate between TS and ACS [40, 41, 42].

TS with neurological triggers appears to present more frequently with heart failure-related dyspnea, rather than chest pain [9]. Patients with SAH are often unable to express cardiac symptoms, therefore cardiac biomarkers, electrocardiography (ECG), and transthoracic echocardiography are essential for the early detection of TS.

### 5.1 Laboratory Tests

**Fig. 1.**
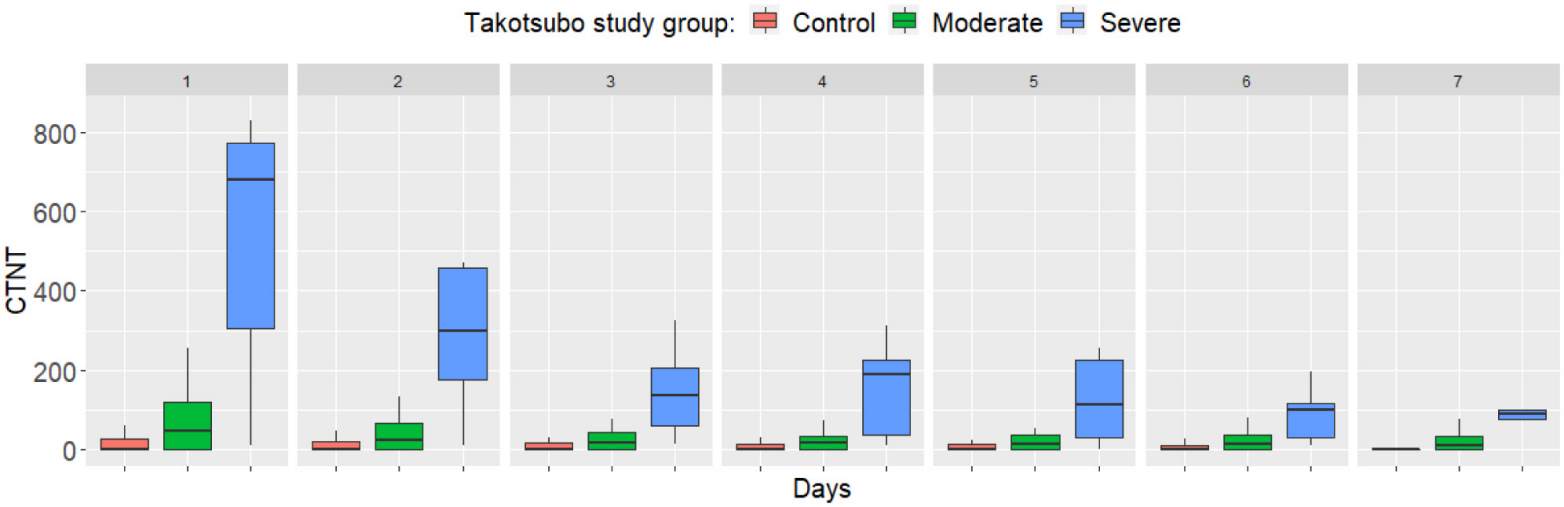
**Differences in cardiac troponin T between study groups from day 1 to 7 after SAH**. Reproduced with permission from Molnár *et al*. [15]. Boxplots show the median, upper, and lower quartiles, and minimum and maximum values, with outliers omitted for clarity. Differences are highly significant each day (*p* < 0.001). CTNT: cardiac troponin T (ng/L); Contol: control SAH patients; Moderate: patients with SAH plus TS, EF >40%; Severe: patients with SAH plus TS, EF <40%. SAH, subarachnoid hemorrhage; TS, Takotsubo syndrome; EF, ejection fraction.

**Fig. 2.**
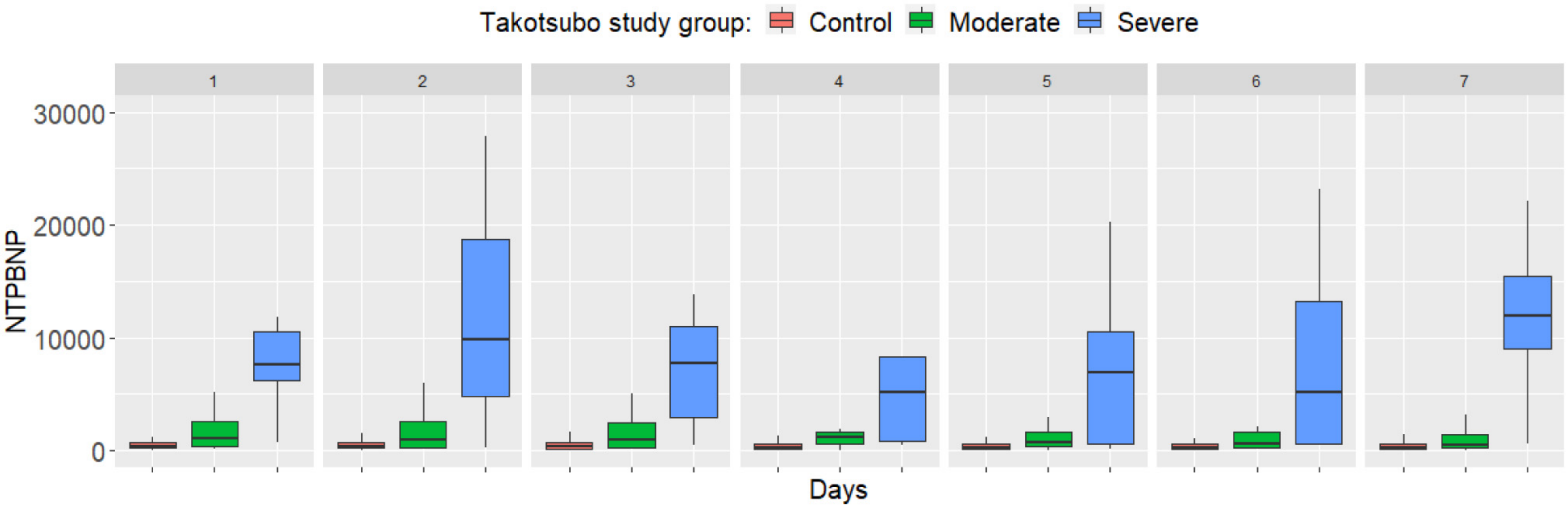
**Differences in N terminal prohormone of brain natriuretic peptide between study groups from day 1 to day 7 after SAH**. Reproduced with permission from Molnár *et al*. [15]. Boxplots show the median, upper, and lower quartiles, and minimum and maximum values, with outliers omitted for clarity. Differences are highly significant each day (*p* < 0.001). NT-proBNP: N terminal prohormone of brain natriuretic peptide (ng/L); Contol: control SAH patients; Moderate: patients with SAH plus TS, EF >40%; Severe: patients with SAH plus TS, EF <40%. SAH, subarachnoid hemorrhage; TS, Takotsubo syndrome; EF, ejection fraction.

Troponin levels are elevated in up to 34% of patients with SAH. Troponinemia correlates with the severity of SAH and the presence of arrhythmias and WMAs [7]. Differently from ACS, in TS the increase in troponin is modest compared to the extent of WMA. The increase in creatine kinase MB (CK-MB) is typically discrete and presents with values lower than those observed in ACS [24, 43]. Significant brain natriuretic peptide (BNP) elevation is common. The N terminal prohormone of brain natriuretic peptide (NT-proBNP) has a considerably longer half-life than the active peptide, thus being more suitable for risk stratification [15, 42]. In patients with TS, high levels of BNP are consistent with the degree of ventricular dysfunction, and this characteristic is different from that seen in patients with ACS. Given these differences, the combination of troponin, CK-MB, and BNP may help differentiate between TS and ACS [24]. Follow-up investigations of cardiac biomarkers in SAH patients showed that cardiac troponin and NT-proBNP values were elevated from the day of admission both in mild (EF >40%) and severe TS (EF <40%) cases. While troponin-T and NT-proBNP levels tended to normalize in mild TS from day 3, in severe TS higher levels of these cardiac biomarkers persisted by day 7 (Figs. 1,2, Ref. [15]). Troponin and BNP have an additional prognostic value, suggesting that higher levels predict a worse outcome [5, 24, 40].

It is widely accepted that catecholamines play a key role in the development of TS, however, serum epinephrine and norepinephrine levels are not routinely measured, because of their short plasma half-life [7]. There is also growing evidence for the pathogenetic role of inflammation, reflected in the fact that the level of C-reactive protein is generally elevated. Interleukin-6, interleukin-7, and a signature of circulating microRNAs (miR-1, miR-16, miR-26a, and miR-133a) are being tested in TS to differentiate early TS from ACS patients [44]. Further investigations are required to determine the diagnostic value of the inflammatory markers mentioned above.

### 5.2 Electrocardiography 

Electrocardiographic changes occur in up to 82% of patients with SAH. Common ECG changes include QTc prolongation, ST-segment changes, T-wave inversions, new Q-waves, abnormal U-waves, sinus bradycardia, and sinus tachycardia [7]. According to Talahma *et al*. [5], in the case of the coexistence of SAH and TS, the most common ECG findings are the following: T-wave inversion, ST depression, QT prolongation, sinus tachycardia, and sinus bradycardia. A prospective study by Kilbourn *et al*. [33] demonstrated that ST elevation is more frequent in patients with TS compared to the non-TS SAH group. ST-segment elevation in the anterior precordial leads can occur in patients with either AMI or TS. In TS, ST elevation usually does not localize to a particular territory [22]. In AMI, ST depression in the inferior leads usually presents as a reciprocal change witch often absent in TS [45]. QT prolongation can be found in a substantial number of cases and can predispose to the risk of developing torsades de pointes tachycardia. The QTc-interval is longer in TS than in AMI. In TS, T-wave inversions are generally widespread and correlate with myocardial edema [22, 46]. Atrioventricular blocks, left bundle branch blocks, and atrial and ventricular fibrillations are not frequent findings [47, 48]. The normalization of myocardial contractile function occurs before long-lasting electrocardiographic abnormalities [48]. Continued prolongation of QTc was associated with an increased risk of in-hospital mortality [40, 49].

### 5.3 Imaging Modalities

Especially in unstable patients, and in the acute phase of SAH echocardiography is the first-line imaging tool for the evaluation of left ventricular function and WMAs. In TS, follow-up echocardiography is recommended daily or every two or three days during the first weeks, and at longer intervals after the acute phase [42, 50]. The main echocardiographic findings include (1) left ventricular WMA, which is independent of epicardial coronary artery distribution in most cases, and (2) reduction in left ventricular EF with improvement in the short term. The classical apical ballooning pattern is commonly associated with basal hyperkinesia, which may lead to dynamic left ventricular outflow tract obstruction (LVOTO), further reducing stroke volume, and is associated with mitral regurgitation due to the systolic anterior motion of the mitral leaflet. The ‘apical nipple sign’ refers to an unaffected region of the ventricular apex that contracts normally, observed in approximately a third of patients with the apical type of TS, and can help distinguish TS from anterior ST-segment elevation myocardial infarction [40]. Atypical morphologic variants include midventricular, basal, and focal TS, generally associated with less reduced left ventricular EF, and appear to be more frequent in acute neurological conditions [24, 51, 52]. The presence of right ventricular involvement is a severity marker for a more eventful clinical course and worse outcomes [40].

Coronary angiography with ventriculography is considered the cornerstone of diagnosis to exclude critical coronary lesions which are the culprits for WMAs. However, patients with SAH are not the best candidates for this procedure, especially in the setting of increased intracranial pressure or untreated aneurysm. According to Murthy *et al*. [7], the following factors promote cardiac catheterization in acute SAH: WMA in a single coronary artery distribution, male sex, elevated troponin level with failure to decay after the first day and low-grade SAH.

Coronary computed tomographic angiography (CCTA) is a noninvasive option with minor risk. It has shown a correlation of 80% when compared with invasive coronary angiography, and the discrepancy in the remaining 20% mainly occurred when minimal non-obstructive atherosclerosis was assessed [13].

Cardiac magnetic resonance imaging (CMRI) can be useful in the acute phase but its use is limited by its availability and technical difficulties in imaging unstable patients. Owing to its ability to detect the presence of edema, a typical finding in TS, CMRI plays a crucial role in ruling out ACS and myocarditis during the post-acute phase. It should be performed in doubtful cases or patients with persistent WMAs, even after discharge [40, 42].

## 6. Monitoring 

In SAH, TS is an underdiagnosed entity. ECG, laboratory testing of cardiac biomarkers, and echocardiography are not routinely performed at admission, which allows several TS cases to remain undetected. Echocardiography is highly recommended in patients with ECG abnormalities and elevated biomarkers. These high-risk patients should be monitored and treated in an intensive care unit. Because of the high incidence of QT prolongation, serial 12-lead ECG should be performed to assess the risk of torsades de pointes tachycardia [19].

Patients with WMA may be asymptomatic or have signs of heart failure. In asymptomatic patients, continuous ECG and invasive blood pressure monitoring may be sufficient. Follow-up echocardiography is recommended in a few days or weeks to verify the resolution of WMA [42]. In the case of symptomatic heart failure, follow-up echocardiography also plays an important role in identifying LVOTO, the systolic anterior motion of the mitral valve, the involvement of the right ventricle, and the severity of left ventricular dysfunction [44]. In the majority of TS cases, recovery of WMAs can be observed within days to months after the acute event. Factors associated with delayed recovery are male sex, left ventricular EF below 45%, and acute neurologic disorders [2, 25]. In accordance with this, studies on SAH patients have demonstrated abnormal wall motion score index (WMSI) on follow-up echocardiograms several weeks after the triggering event, especially in patients with low initial EF [15, 33].

The use of invasive hemodynamic monitoring, such as Pulse index Contour Continuous Cardiac Output (PiCCO), may be beneficial in cardiogenic shock or pulmonary edema. Advanced transpulmonary thermodilution techniques can help in guiding fluid and inotropic therapy and maintain proper cerebral perfusion [53, 54].

## 7. Treatment 

There are no official guidelines for the management of TS in SAH. Randomized controlled trials are lacking and the current therapy is mainly based on expert opinions.

### 7.1 Beta-Blockers

According to catecholamine theory, beta-blocker therapy may be beneficial. Beta-blockers not only have an adrenergic blocking effect but are also cerebroprotective as they decrease cerebral metabolism. In a retrospective study by Liang *et al*. [37], prehospital administration of beta-blockers lowers the risk of TS after SAH. However, in another single-center retrospective study, beta-blocker therapy was not associated with better neurological outcomes in SAH patients with TS [5]. Early beta-blocker therapy also failed to reduce 30-day mortality [55]. In patients with severe LV dysfunction, beta-blockers are used as standard therapy for heart failure. When heart failure is combined with LVOTO β-1 selective beta-blockers are recommended, while for patients without LVOTO, carvedilol is the best choice [16, 56].

### 7.2 Positive Inotropes 

In general, all types of catecholamines should be avoided as they may aggravate the excessive catecholamine release and cause microvascular dysfunction and coronary artery spasm [5]. On the other hand, when cerebral vasospasm develops, it is essential to elevate cardiac output and increase cerebral perfusion.

In severe heart failure, the use of non-catecholamine inotropes can promote hemodynamic stability [5, 57]. However, experimental studies have shown that milrinone may induce apical ballooning in animal models [16]. Milrinone is a phosphodiesterase inhibitor with inotropic and vasodilating properties. Its use is controversial, as it may improve contractility and cardiac output, but may also induce hypotension through its vasodilator effect. A retrospective observational study showed that milrinone increases cardiac index in patients with TS. If TS is combined with severe cerebral vasospasm, the use of milrinone can improve cerebral perfusion and reduce the risk of delayed cerebral ischemia [13]. Other studies have shown that milrinone may decrease systolic blood pressure, therefore its use is recommended only if the systolic blood pressure is above 90 mmHg [58].

Levosimendan is a new type of calcium sensitizer, with an inotropic effect, that improves cardiac output. It also has a vasodilator effect, decreases preload, and improves coronary perfusion and WMAs. Therefore, it is a promising therapeutic option for patients with or without cerebral vasospasm [44, 48, 59].

In the case of LVOTO vasodilators, diuretics, and positive inotropes should be avoided [25, 56].

### 7.3 Angiotensin-Converting Enzyme Inhibitors (ACEIs)/Angiotensin Receptor Blockers (ARBs)

ACEIs and ARBs are part of the standard therapy for heart failure. A recent meta-regression study showed that TS patients on ACEI/ARB treatment at discharge had a better survival rate and lower risk of recurrence of stress cardiomyopathy [60]. ACEI/ARB monotherapy or its combination with beta-blockers has a role in the prevention of TS recurrence during long-term follow-up [61].

### 7.4 Insulin

Cathecolamine-induced insulin resistance is a well-known phenomenon. Insulin not only lowers the serum glucose level but also has several pleiotropic effects. It increases contractility, cardiac output, and glucose uptake in the stunned myocardium [48]. In a case report of Chandler *et al*. [62], a rapid improvement in stroke volume was achieved with hyperinsulinemic euglycaemic therapy.

### 7.5 Adenosine

Intravenous administration of adenosine has a potent anti-catecholaminergic effect, decreases norepinephrine levels, and may improve myocardial perfusion and WMAs [16].

### 7.6 Mechanical Support

For patients with refractory hypotension secondary to severe heart failure, mechanical support should be considered [63]. In cases of LVOTO the use of a left ventricular assist device (LVAD), Impella is recommended. LVAD, Intra-aortic balloon pump (IABP), or veno-arterial extracorporeal membrane oxygenation (VA-ECMO) may improve stroke volume in severe heart failure without LVOTO [48, 64, 65].

### 7.7 Arrhythmias

As a result of elevated catecholamine levels and myocardial inflammation, there is a high risk of arrhythmias in the acute phase of TS. ECG monitoring has the utmost importance in rapid diagnosis and treatment. QT-prolonging drugs must be withdrawn to avoid life-threatening arrhythmias [19]. The use of implantable pacemakers and cardiac defibrillators in the event of life-threatening arrhythmias is a matter of debate. Within some weeks or months, the risk of arrhythmias decreases with the resolution of TS, therefore, in most cases, only temporary treatment is needed [9, 16].

### 7.8 Anticoagulation

In severe TS with typical apical ballooning, ventricular thrombus may develop. Heparin is recommended to decrease the risk of thromboembolism only after treatment of the aneurysm. For patients with intraventricular thrombus or thromboembolism, at least three months of anticoagulation therapy is required [48, 66, 67, 68].

## 8. Outcome

Previously, TS was thought to have a good prognosis due to its reversibility. However, recent studies have reported in-hospital mortality of 3.5–10.6%, comparable to that of ACS [25, 69, 70]. Its recurrence rate is estimated to range from 2% to 5% [70, 71]. Physical trigger factors, especially acute neurological disorders, are predictors of adverse outcomes. Although the focal form of TS has a more favourable outcome, biventricular involvement is a severity marker associated with a worse prognosis [40]. In SAH, TS is associated with an increased risk of cardiac and noncardiac complications. Previous reports suggest that TS is a risk factor for arrhythmias, pulmonary edema, and delayed cerebral ischemia in SAH [3, 54, 72, 73]. The appearance and severity of TS have been associated with higher mortality and worse clinical outcomes [3, 5, 15, 33, 72, 74]. A recent prospective follow-up study demonstrated unfavourable functional status according to the Barthel Index, the Karnofsky Scale, and Glasgow Outcome Scale at 30 and 180 days in TS patients with EF <40% compared to the control non-TS SAH group [15]. Since TS occurs most frequently in massive SAH, the severity of the neurogenic injury has a pronounced effect on the overall prognosis of these patients [7]. Cardiac complications are generally not the main reasons for fatal outcomes [9].

## 9. Discussion

Recent prospective studies have confirmed that TS is a relatively common complication of acute SAH, occurring in up to 28% of cases [2]. Retrospective studies have shown a much lower incidence, indicating the high probability of underdiagnosis of TS [5, 30, 34, 35]. On the other hand, the clinical presentation of TS is similar to that of AMI, which increases the risk of misdiagnosis. Differential diagnostic issues and hemodynamic instability resulting from TS can delay the definitive treatment of the aneurysm. In addition, a decrease in MAP secondary to heart failure and a consequent decrease in cerebral perfusion may contribute to further neuronal damage, especially in the setting of increased intracranial pressure. Therefore, rapid diagnosis and hemodynamic stabilization are key points in acute care. In those cases, when cerebral vasospasm and TS are present together, reaching the target MAP required for adequate cerebral perfusion is challenging, putting these patients at increased risk of delayed cerebral ischemia [54]. Therapeutic agents that reduce exogenous catecholamine intake may be beneficial, contributing to earlier recovery of ventricular function [5].

The relatively high incidence of TS and its significant effect on the cerebral circulation draw attention to the importance of screening at admission, as well as in all cases of acute deterioration during hospitalization (e.g., vasospasm, rebleeding, acute hypotension) [75]. ECG, cardiac biomarkers, and echocardiography are widely available bedside methods that are suitable for screening and help differentiate TS from AMI [7, 13]. However, none of the ECG changes is specific for TS, prolongation of the QTc interval can be found in a substantial number of TS cases, and the QTc interval is longer in TS than in AMI. QTc prolongation predisposes to the development of torsades de pointes tachycardia, indicating a need for advanced monitoring [35, 40, 49]. Widespread T-wave inversion is also a common finding in TS [22, 35]. Unlike ACS, in TS, the increase in troponin is modest, the increase in CK-MB is usually discrete, and the high levels of NT-proBNP are consistent with the degree of ventricular dysfunction. Given these differences, the combination of troponin, CK-MB, and NT-proBNP may help differentiate between TS and ACS [15, 24, 42, 43]. Echocardiography is the first-line imaging tool for the evaluation and follow-up of left ventricular function and WMA. In acute neurological conditions, atypical TS morphology (basal, midventricular, and focal) seems to be more frequent [24]. Distinguishing the focal form from coronary artery disease (CAD) and AMI is the most challenging, but cardiac biomarkers, changes in the ECG, and the presence of risk factors (TS is more common in women and in high-grade SAH, cardiovascular risk factors are more characteristic of CAD and AMI) can provide help [9]. In general, coronary angiography with ventriculography is the cornerstone of the diagnosis of TS to exclude critical coronary lesion which is responsible for WMA [31, 76]. In SAH, the benefits of this invasive diagnostic method are questionable. Before securing the aneurysm, there is a high risk of rebleeding and the administration of anticoagulant and antiplatelet agents required for coronary intervention should be avoided. Coronarography, performed only for diagnostic purposes, has few benefits and also delays the definitive treatment of the aneurysm. Successful endovascular treatment of the aneurysm allows the administration of anticoagulants and antiplatelet agents for coronary intervention [77]. However, further neurosurgical procedures may become necessary in the following days and weeks (e.g., placement of an external ventricular drain or ventriculoperitoneal shunt), which require the reversal of anticoagulation and antiplatelet therapy, even if a coronary stent was previously inserted, placing these patients at high risk of stent thrombosis.

With regard to the above, the authors of this article suggest that acute coronary angiography should be considered after successful endovascular treatment of the aneurysm in patients with strong evidence of myocardial ischemia (WMA in a single coronary artery distribution, high troponin and CK-MB level with moderate elevation of NT-proBNP, ECG changes characteristic of AMI, male sex, low-grade SAH, presence of cardiovascular risk factors), when coronary intervention may improve left ventricular function and contribute to hemodynamic stability. The risk-benefit ratio must be considered individually in each patient. In other doubtful cases, CCTA or CMRI serves as a noninvasive option with lower risk [40, 42]. The timing of these examinations depends on the general condition of the patient.

**Fig. 3.**
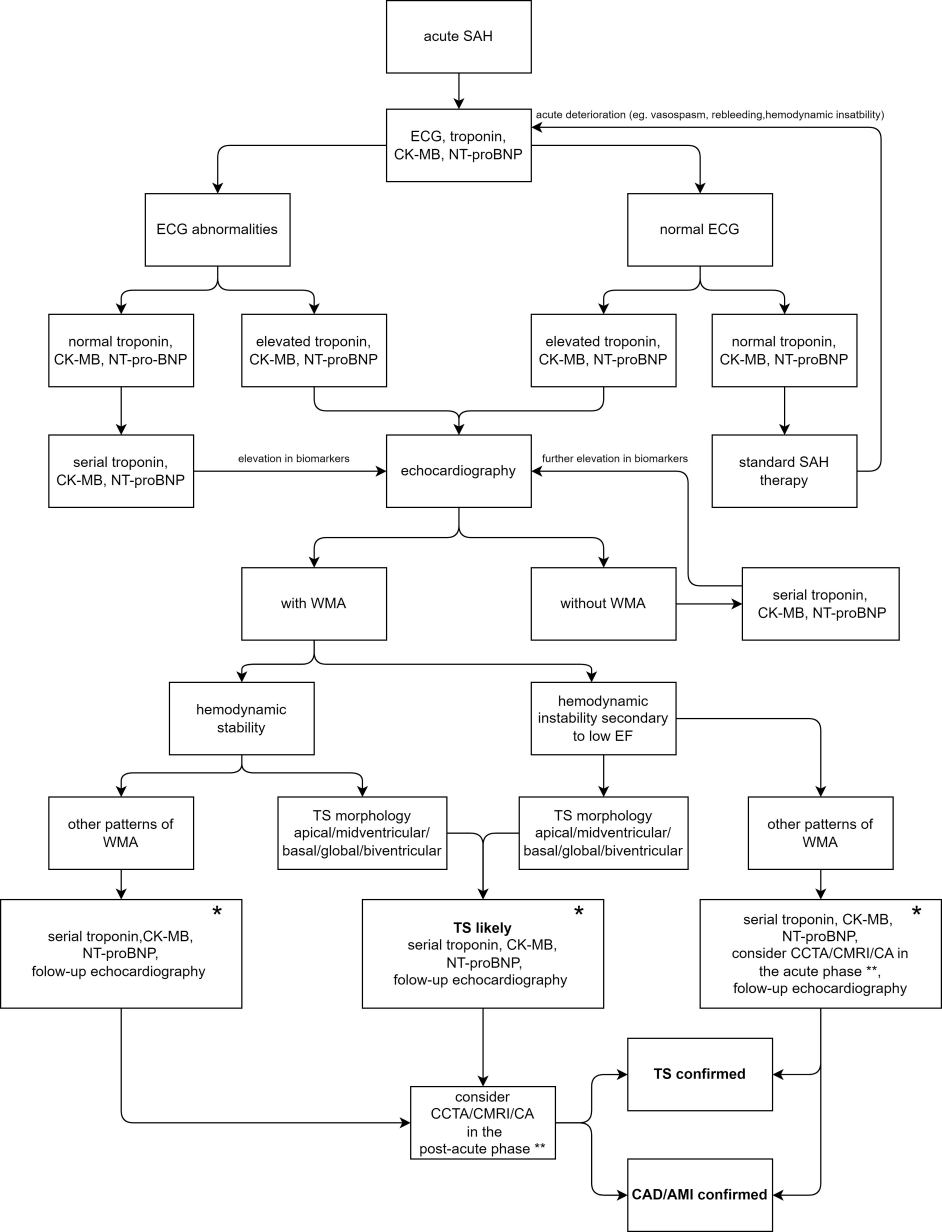
**Diagnostic algorithm for detection of Takotsubo syndrome in patients presenting with subarachnoid hemorrhage**. TS, Takotsubo syndrome; SAH, subarachnoid hemorrhage; ECG, electrocardiography; CK-MB, creatinin kinase MB; NT-proBNP, N terminal prohormone of brain natriuretic peptide; WMA, wall motion abnormality; EF, ejection fraction; CAD, coronary artery disease; AMI, acute myocardil infarction; CCTA, coronary coputed tomography angiography; CMRI, cardiac magnetic resonance imagining; CA, coronary angiography. * AMI is more likely to be present in the following cases: RWMA in a single coronary artery distribution, male sex, elevated troponin level with failure to decay after the first day, and low-grade SAH [7]. The combination of troponin, CK-MB, and NT-proBNP, ECG changes and the reversibility of WMA may also help differentiate between TS and AMI [24, 42]. In contrast to myocardial infarction, age, and cardiovascular risk factors do not predispose to the development of TS [15, 33, 34, 35, 38]. **Coronary angiography is indicated in only those cases when the patient may benefit from the coronary intervention, considering the necessity and risk-benefit ratio of anticoagulant therapy.

If there is a high probability of TS based on ECG, cardiac biomarkers, echocardiography, and risk factors, CMRI/CCTA/coronary angiography (CA) can be postponed to the post-acute phase (Fig. 3, Ref. [7, 15, 24, 33, 34, 35, 38, 42]). Even in these patients, securing the aneurysm is the priority; in the case of hemodynamic instability, the endovascular route is preferred [27, 78]. In TS, close follow-up with echocardiography is recommended in the acute and post-acute phases [42]. Reversal of wall motion abnormalities can be observed within days to months, delayed recovery is more frequent in patients with low initial EF [15, 33].

**Fig. 4.**
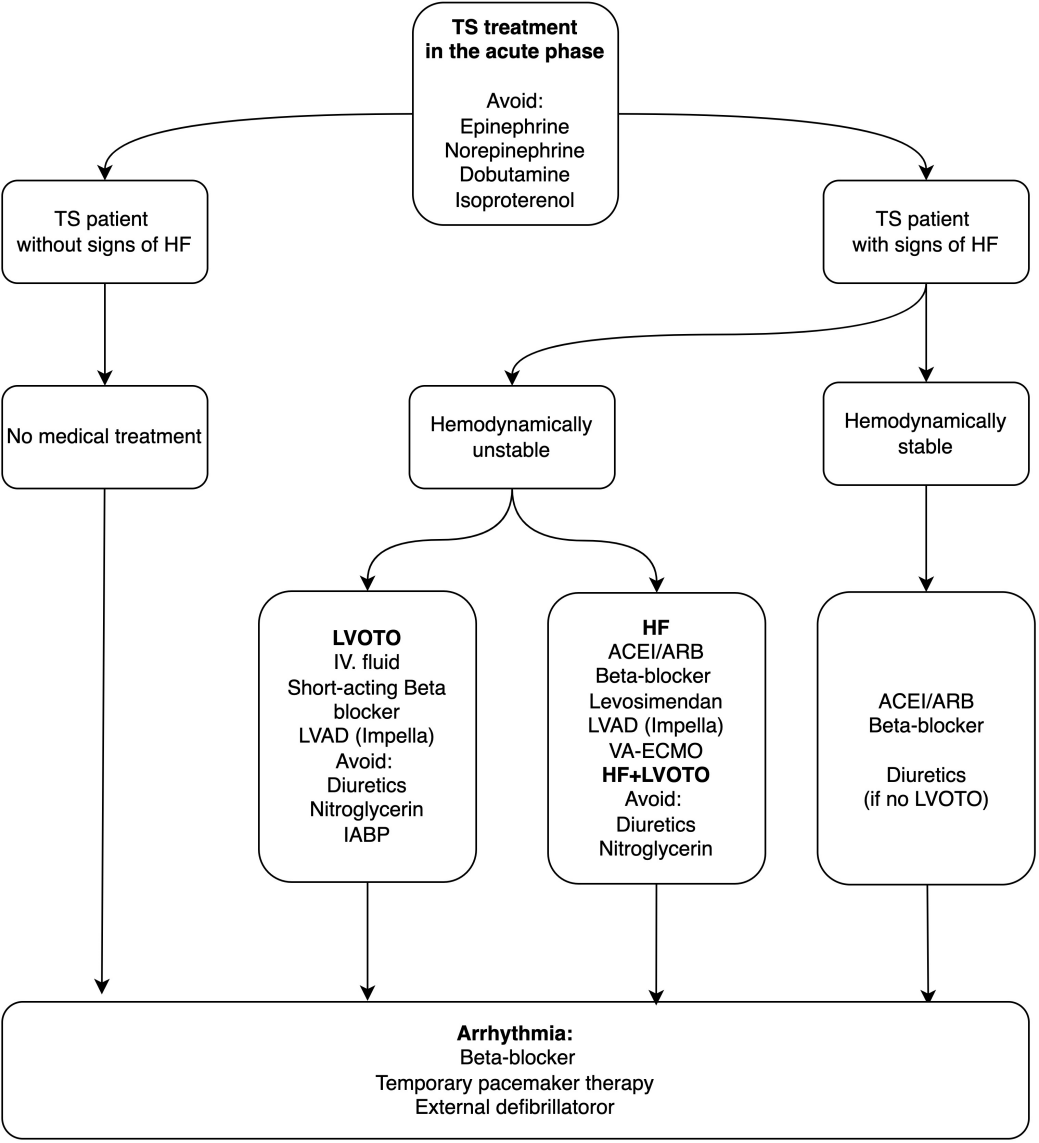
** Management algorithm of Takotsubo Syndrome in the acute phase of subarachnoid hemorrhage**. ACEI, angiotensin-converting enzyme inhibitor; ARB, angiotensin receptor blocker; HF, heart failure; IABP, intra-aortic balloon pump; IV, intravenous; LVAD, left ventricular assist device; LVOTO, left ventricular outflow tract obstruction; TS, Takotsubo syndrome; VA-ECMO, venoarterial extracorporeal membrane oxygenation.

The aim of TS therapy in SAH is twofold: maintenance of adequate cerebral perfusion to avoid further neurological damage and promote recovery of the myocardium. Administration of exogenous catecholamines may contribute to the expansion of WMA and delay the recovery of the left ventricular function, thus catecholamines should be avoided or used with great caution [5]. In asymptomatic patients with mild left ventricular dysfunction, no specific therapy is required. For symptomatic patients, standard heart failure therapy is recommended (ACEI/ARB, beta-blockers, and diuretics). In hemodynamically unstable patients, the use of non-catecholamine inotropes, especially levosimendan, may be advantageous [57, 59, 79]. Invasive hemodynamic monitoring, such as PiCCO, may be beneficial in cases of cardiogenic shock or pulmonary edema [53, 54]. In patients with refractory hypotension, mechanical support should be considered [63]. In the case of LVOTO vasodilators, diuretics, positive inotropes, and IABP should be avoided [25, 56]. The recommended therapy for patients with LVOTO is intravenous fluid and short-acting beta-blockers, and in refractory cases, LVAD insertion can contribute to hemodynamic stability [56, 79]. There is a high risk of arrhythmias in the acute phase of TS. The administration of beta-blockers, placement of a temporary pacemaker or an external defibrillator may become necessary [9, 16, 39]. Drugs prolonging QT-interval must be withdrawn to avoid torsades de pointes tachycardia [19] (Fig. 4). ACEIs or ARBs with or without beta-receptor blocking agents may play a role in the prevention of TS recurrence [80].

In SAH, the occurrence and severity of TS have been associated with higher mortality and worse clinical outcome [3, 5, 15, 33, 72]. However, the severity of neurogenic injury has a major effect on the overall prognosis and cardiac complications are generally not the main, determining causes of fatal outcomes [7, 9, 81]. In most cases of TS, spontaneous recovery of left ventricular function can be observed within days to months after the acute event [2].

## 10. Conclusions

TS is an underdiagnosed entity in SAH, however, it may have a major effect on cerebral circulation and predicts poor outcomes. Therefore, screening for TS at admission and in cases of acute deterioration is warranted. Noninvasive bedside diagnostic methods are preferred to detect TS and to distinguish it from AMI. Aneurysm securing should have priority over coronary angiography in the acute phase. Rapid hemodynamic stabilization and early aneurysm treatment are essential to reduce the risk of rebleeding and prevent delayed cerebral ischemia, which may contribute to a more favourable outcome.
